# Fabrication of Low-Cost and Customizable Planar Electrochemical Devices Using Multi-Material 3D Printing and Platinum Leaves

**DOI:** 10.3390/s26144528

**Published:** 2026-07-16

**Authors:** Michele Abate, Gino Bontempelli, Nicolò Dossi

**Affiliations:** Sustainable Analytical Instrumentation Laboratory (Sustain Lab), Department of Agricultural, Food, Environmental and Animal Science, University of Udine, Via Cotonificio 108, I-33100 Udine, Italy; michele.abate@uniud.it (M.A.); gino.bontempelli@uniud.it (G.B.)

**Keywords:** 3D-printed electrochemical device, portable analytical devices, metal leaf electrode, platinum gold leaf, polyester adhesive, polycaprolactone, hydrogen peroxide

## Abstract

This paper introduces a novel method for producing planar electrochemical devices by combining multi-material 3D printing with metal leaves (3D-MLEs). The fabrication process is based on the use of two polymeric materials, polylactic acid (PLA) and polycaprolactone (PCL), leveraging their different melting points. The approach exploits the thermoadhesive properties of polyesters, which can act as bonding layers upon heating, enabling a direct-writing and low-step fabrication strategy. The device was fabricated using a dual-extruder 3D printer to produce a PLA support containing PCL tracks, followed by selective thermal adhesion of the metal leaf onto the PCL. This process exploits the different melting temperatures of the two polymers: PCL softens and becomes adhesive at the selected temperature, while the PLA support remains structurally unaffected. A final brushing step enables the definition of a well-controlled three-electrode geometry. Following optimization of the fabrication parameters, a platinum leaf-based device (3D-PtLE) was assembled and evaluated using potassium hexacyanoferrate(II) and hexaammineruthenium(III) chloride as redox probes. The optimized device was subsequently applied to hydrogen peroxide detection in phosphate buffer (pH 7), exhibiting a linear response in the concentration range of 0.25–5 mM, with a limit of detection of 67 μM and good repeatability (RSD = 4.2%). The analytical applicability of the device was further demonstrated through the analysis of a real sample consisting of washing water prepared from a sodium percarbonate-based cleaning tablet, with good agreement (98 ± 6%) with the standard titration method. The proposed strategy provides a simple, low-cost, and customizable approach for fabricating planar electrochemical platforms based on pure metal electrodes, combining high analytical performance with straightforward manufacturing.

## 1. Introduction

In recent decades, the growing focus on environmental, economic, and social sustainability has also influenced analytical chemistry. This evolution has led to the development of new analytical approaches and instrumentation in agreement with the fundamental principles established by Green Analytical Chemistry (GAC) and Democratic Analytical Chemistry (DAC) [1].

The scientific literature reports several examples of low-cost analytical devices based on these principles, including paper- and plastic-based colorimetric platforms, microfluidic systems, unconventional conductive materials for electrochemical measurements and smartphone-integrated platforms [2,3,4,5,6,7,8,9,10]. The integration of sensing elements with automated microfluidics further represents a promising strategy for achieving reliable on-site analysis through improved sample handling and reduced operator intervention [11]. Electrochemical techniques are particularly well suited for the development of sustainable analytical instrumentation, as they meet the requirements of GAC and DAC. In particular, the use of three-electrode electrochemical cells in a planar configuration offers several operational advantages, including portability, low energy consumption, limited use of reagents and solvents, and reduced waste generation [12]. Carbon-based materials, such as graphite, graphene, carbon black, and carbon nanotubes, are the most common choices for electrode fabrication. These substances can be incorporated into conductive ink formulations that are subsequently applied using substrate deposition techniques, such as screen printing or inkjet printing [13,14,15,16].

An emerging alternative is 3D printing, which has been proposed for obtaining individual electrodes or entire electrode circuits with customized geometries. In particular, the Fused Deposition Modeling (FDM) technique enables the layer-by-layer deposition of molten thermoplastic materials. Conductive filaments used for this purpose typically consist of thermoplastic polymers (such as PLA or ABS) containing carbonaceous conductive materials. The main strategic advantage of 3D printing lies in its ability to easily modify electrode and cell geometries, allowing for the fabrication of devices tailored to specific electroanalytical requirements [17,18,19,20,21,22]. Although carbon-based electrode materials are widely used due to their availability and low-cost, some specific electrochemical applications require metal electrodes such as gold, platinum, copper, palladium, silver, or iridium. These metals are exploited for their electrocatalytic properties, often related to the spontaneous or electrochemically induced formation of metal oxides on their surfaces [23,24].

The manufacture of planar metal electrodes using conventional procedures, such as Chemical Vapor Deposition (CVD) or Physical Vapor Deposition (PVD), requires clean-room facilities and technologically complex and expensive equipment [25,26].

More recently, 3D-printed metal electrode platforms have also been explored, including metallic structures obtained through advanced manufacturing techniques such as selective laser melting (SLM) [27] and polymer-based electrodes containing metallic fillers [28,29,30,31]. Although these approaches may provide enhanced electrochemical performance, they often require expensive instrumentation, complex post-processing steps, or composite materials that may exhibit heterogeneous electrochemical behavior and limited reproducibility.

As a more accessible alternative, screen-printed electrodes (SPEs) based on inks containing metals, nanoparticles, or metal oxides have been proposed. However, the ink matrix may contain components capable of acting as interferents, introducing variability in electrode performance [32,33,34,35]. Another strategy for obtaining metallic surfaces involves the electrodeposition of metal ions onto carbon-based electrodes, including SPEs and 3D-printed devices, to enhance their electrocatalytic properties. However, this approach requires careful optimization of deposition parameters and may generate metal-containing waste, partially conflicting with GAC principles [36,37]. Therefore, despite significant advances in both polymer-based and metal-based 3D-printed electrodes, a trade-off still exists between fabrication simplicity, material purity, and electrochemical performance.

Metal leaves, consisting of thin layers of pure metals such as gold, platinum, or copper, represent a low-cost and easily handled alternative to fabricating planar metal electrodes. Their high purity, electrical conductivity, and reflectivity make them suitable for simultaneous electroanalytical and spectroelectrochemical applications. However, their integration onto rigid supports often requires adhesives, which may introduce interferences and affect analytical reliability [38,39,40].

Despite recent advances, a simple and sustainable strategy capable of combining the geometric flexibility of additive manufacturing with the intrinsic conductivity, purity, and reproducibility of bulk metal electrodes is still lacking. To address these challenges, we propose a fabrication strategy exploiting the thermoadhesive properties of polyester-based commercial filaments to integrate metal leaves with multi-material 3D printing (3D-printed Metal Leaf Electrodes, 3D-MLEs), enabling the fabrication of low-cost planar platinum three-electrode electrochemical cells (3D-PtLEs). This approach allows independent optimization of electrode geometry and electroactive material, avoiding conductive inks, adhesives, nanoparticle incorporation, or vacuum-based deposition techniques.

Following a preliminary evaluation of its analytical performance, conducted using potassium ferrocyanide and hexaammineruthenium chloride as redox probes, the device assembled using platinum leaves was used to determine hydrogen peroxide in neutral aqueous solutions as a proof of concept. Hydrogen peroxide was selected as a benchmark analyte due to its sensitivity to platinum surface properties and its widespread use in evaluating Pt electrocatalytic behavior. In addition, the determination of hydrogen peroxide is of great importance in several fields, including the food and medical sectors [41,42,43].

As a proof-of-concept application to a real sample, the optimized device was applied to the analysis of residual hydrogen peroxide in washing solutions prepared from sodium percarbonate-based tablets, which are commonly used for cleaning and sanitizing small kitchen appliances.

## 2. Materials and Methods

### 2.1. Chemicals

Potassium hexacyanoferrate(II) (K_4_[Fe(CN)_6_], ≥99.0%), hexamineruthenium(III) chloride ([Ru(NH_3_)_6_]Cl_3_), potassium chloride (KCl), sulfuric acid (H_2_SO_4_, 95%), nitric acid (HNO_3_, 65%), potassium dihydrogen phosphate (KH_2_PO_4_, ≥98%), potassium monohydrogen phosphate (K_2_HPO_4_, ≥98%) and hydrogen peroxide (H_2_O_2_, 30%) were purchased from Sigma-Aldrich (Sigma-Aldrich, Milano, Italy).

Deionized water obtained with a Purelab Flex 3 system (Elga, High Wycombe, UK) was used to prepare aqueous solutions. These consisted of a 0.1 M sulfuric acid solution, a 1:10 (*v*/*v*) concentrated nitric acid solution, and a 0.1 M phosphate buffer at pH 7, measured with a pH meter (Crison pH-2001 Hach Lange, L’Hospitalet de Llobregat, Spain). The standard solutions of potassium hexacyanoferrate(II) and hydrogen peroxide were prepared in deionized water to obtain final concentrations of 10 mM and 100 mM, respectively, and were diluted when necessary to the desired concentration with the appropriate electrolyte solution.

3D-printed Metal Leaf Electrodes (3D-MLEs) were assembled using platinum (3D-PtLEs) or 24-carat gold leaves (3D-AuLEs) (AP Verona, Verona, Italy), polycaprolactone (PCL) (3D4Makers, Haarlem, The Netherlands) and polylactic acid (PLA) (FiloAlfa, Torino, Italy) filaments.

### 2.2. Fabrication of 3D-MLEs

The geometry of the 3D-MLE cells was inspired by that of commercial screen-printed electrodes, consisting of a working electrode (12.6 mm^2^) (WE), a pseudo-reference electrode (PRE), and a counter electrode (CE). The design was created using the Autodesk Fusion 360 software v. 2702.1.47 (Autodesk, San Rafael, CA, USA).

**Figure 1:**
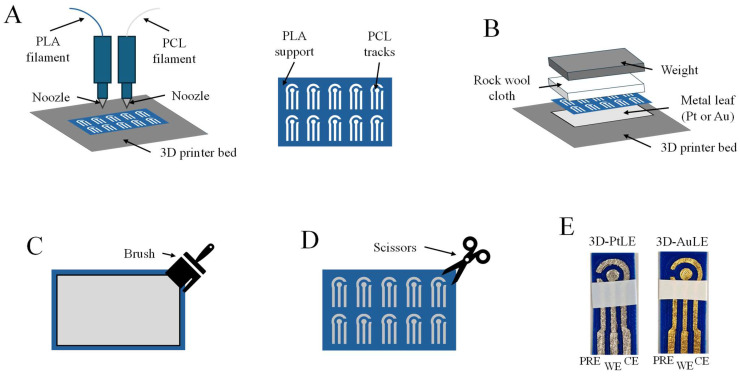
Scheme of the assembly procedure of the 3D-MLEs (**A**–**D**) and picture of the assembled devices (**E**).

A series of ten electrochemical cells with the configuration described above was assembled using a FLSUN Cube 3D printer (Zhengzhou Chaokuo Electronic Technology Co., Zhengzhou City, China), equipped with a dual filament extruder. The printing conditions used for the polylactic acid (PLA) support (14.5 × 3.5 × 0.8 mm; L × H × W) were as follows: nozzle diameter of 0.4 mm; printing speed of 50 mm·s^−1^; layer height of 0.2 mm; and extrusion and print bed temperatures of 180 °C and 65 °C, respectively. The electrode circuit traces were printed using a PCL filament. In this case, the printing conditions were nozzle diameter of 0.4 mm; printing speed of 50 mm·s^−1^; layer height of 0.2 mm; and extrusion and print bed temperatures of 80 °C and 65 °C, respectively (Figure 1A).

After assembly, the electrode series underwent a cleaning treatment by immersion in an isopropanol solution to remove surface residues and traces of the adhesive used on the printing bed, ensuring a perfect adhesion interface between the metal leaf and the PCL trace.

The metal leaf was applied using the heat generated by the printer bed of the 3D printer itself. The application of the metal leaf on the printer bed was conducted by placing the metal leaf onto the printer bed and positioning the device substrate on top of it, ensuring direct contact between the PCL electrode trace and the metal. The printer bed was then heated to 60 °C for 20 min and left to cool at room temperature before removing the devices. To allow optimal contact and maintain a stable temperature, the system was thermally insulated using a rock wool cloth and subjected to uniform pressure by applying a metal weight (approximately 50 g) (Figure 1B).

Under the operating conditions described above, PCL behaved as a thermoplastic bonding agent between the PLA substrate and the metal leaf. This capability has already been reported in the scientific literature, demonstrating that polyester-based materials are capable of ensuring strong interfacial adhesion between two surfaces, thanks to their controlled melting properties [44,45,46]. In this context, by operating above the melting point of PCL but below that of PLA, more than satisfactory adhesion between the substrate and the metal was established.

Finally, the excess portion of the metal leaf was removed by brushing (Figure 1C). The individual electrochemical cells were then separated (Figure 1D), and the electrode area was delimited using insulating adhesive tape (area 1.4 × 0.7 mm).

Using this procedure, it was possible to create different types of electrochemical cells using leaves made of different metals. Figure 1E shows two types of devices, one using a gold leaf (3D-AuLE) and the other using a platinum leaf (3D-PtLE). In this study, all electrochemical measurements reported were performed with the platinum leaf-based cell (3D-PtLE).

### 2.3. Voltammetric Measurements at 3D-PtLEs

All voltammetric measurements were carried out using a UV/Vis SPELEC 200–900 nm instrument (Metrohm-DropSens, Varese, Italy) controlled by DropView SPELEC software v. 3.2.2.

Electrochemical optimization of the redox probe was conducted in a conventional electrochemical cell with a total volume of 15 mL. The cell setup included a platinum wire as the counter electrode and a Ag/AgCl (3 M KCl) reference electrode. This configuration enabled the insertion of a platinum disc working electrode (Ø = 2 mm, CH Instruments, Austin, TX, USA) into the same electrochemical cell, allowing comparative measurements to be performed after it was mechanically polished with alumina.

Voltammetric determination of hydrogen peroxide was performed by depositing a small volume of sample (80 μL) directly onto the 3D-PtLE, which was placed horizontally on a planar surface (drop mode).

The electrochemical behavior of the surface was studied by using 1 mM K_4_[Fe(CN)_6_] dissolved in a 0.1 M phosphate buffer at pH 7 as the supporting electrolyte. This analyte was chosen as a prototype redox analyte due to its known reversible electrochemical behavior. Cyclic voltammetry was performed in a potential range between 0 V and +0.5 V vs. an Ag/AgCl, 3 M KCl reference electrode, with a scan rate of 50 mV·s^−1^. Before measurements, each device was pretreated using the following procedure: (i) cleaning with a 1:10 (*v*/*v*) solution of concentrated HNO_3_ and water; (ii) treatment with 30 cyclic voltammograms in 0.5 M H_2_SO_4_ in the range between −0.4 V and +1.25 V; and (iii) treatment with 100 cyclic voltammograms with 0.1 M phosphate buffer at pH 7 in the range between 0 V and +0.5 V with a scan rate of 100 mV·s^−1^.

Hydrogen peroxide was determined using linear sweep voltammetry (LSV) in the potential range between +0.3 and +1.1 V (vs. Ag/AgCl, 3 M KCl) with a scan rate of 100 mV·s^−1^. A 0.1 M phosphate buffer at pH 7 was used as the supporting electrolyte [47]. Before each analysis, the electrode immersed in the sample was pretreated as follows: (i) conditioning at −0.4 V for 60 s; (ii) treatment with 15 cyclic voltammograms in the range between 0 V and +0.5 V with a scan rate of 100 mV·s^−1^.

### 2.4. Real Sample Preparation

Cleaning tablets for coffee machines, consisting of sodium percarbonate (with a content between 10 and 30% *w*/*w*), were purchased from a local retailer. The sample was prepared by simulating the operating conditions of a coffee machine cleaning protocol. Specifically, a single tablet (1.5 g) was dissolved in 300 mL of deionized water previously heated to a temperature of 95 °C and kept under constant agitation. After cooling to room temperature, the solution obtained was diluted at a ratio of 1:10 (*v*/*v*) using phosphate buffer at pH 7. This sample was directly analyzed by voltammetry.

## 3. Results and Discussion

### 3.1. Electrochemical Characterization of 3D-PtLEs

The electrochemical performance of the assembled device was assessed using K_4_[Fe(CN)_6_] as a redox probe to evaluate the electron transfer kinetics at the electrode surface. The probe was prepared at a concentration of 1 mM in a 0.1 M phosphate buffer solution (pH 7). Measurements were conducted in a conventional electrochemical cell with a total volume of 15 mL. The cell setup included a platinum wire as the counter electrode and an Ag/AgCl (3 M KCl) reference electrode to ensure stable reference potential.

**Figure 2:**
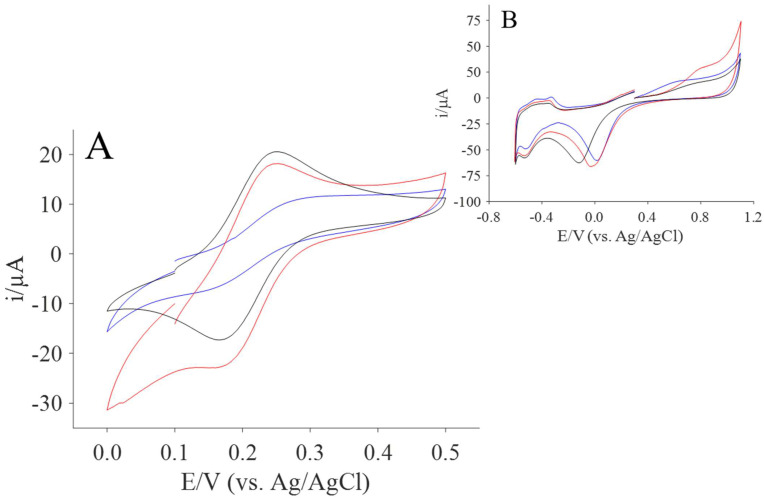
(**A**) Cyclic voltammograms of 1 mM of K_4_[Fe(CN)_6_] in 0.1 M phosphate buffer (pH 7) recorded at a newly fabricated 3D-PtLE pretreated with a solution of concentrated HNO_3_ and water (1:10 *v*/*v*) (blue line), after electrochemical cleaning with H_2_SO_4_ 0.5 M (red line) and after treatment with 100 cyclic voltammetries in 0.1 M neutral phosphate buffer (black line) vs. an external Ag/AgCl, 3M KCl. Scan rate 50 mV·s^−1^. Inset (**B**): Cyclic voltammograms background recorded on phosphate buffer 0.1 M (pH 7) at a newly fabricated 3D-PtLE pretreated with a solution of concentrated HNO_3_ and water (1:10 *v*/*v*) (blue line), after electrochemical cleaning with H_2_SO_4_ 0.5 M (red line) and after treatment with 100 cyclic voltammetries in 0.1 M neutral phosphate buffer (black line) vs. an external Ag/AgCl, 3M KCl. Scan rate 50 mV·s^−1^.

Prior to testing, the newly fabricated device underwent a pretreatment step involving immersion in a solution of concentrated nitric acid and water (1:10 *v*/*v* ratio) for 10 s. This step was intended to eliminate macroscopic contaminants and metallic residues from the manufacturing process. Nonetheless, as shown in Figure 2A, cyclic voltammograms recorded within the potential range from 0 to +0.5 V (vs. Ag/AgCl, 3 M KCl) displayed poor reversibility and limited reproducibility. These results were attributed to a slow electron transfer caused by surface impurities that remained adsorbed during fabrication in an uncontrolled environment. This surface fouling presented a significant challenge for thin-film electrodes, where abrasive mechanical polishing is not viable due to the risk of structural damage of the metallic layer.

To mitigate these challenges, an electrochemical activation protocol was introduced. This involved executing 30 cycles of cyclic voltammetry between −0.4 and +1.25 V (vs. Ag/AgCl, 3 M KCl) at a scan rate of 50 mV·s^−1^ in a 0.5 M sulfuric acid solution. The activation mechanism is consistent with well-established platinum electrochemistry. Potential cycling in sulfuric acid induces repeated formation and reduction in platinum oxide species, which can promote surface cleaning and contribute to the electrochemically active surface area improvement [48,49]. Although the treatment led to an improvement in the voltammetric response of the ferrocyanide probe, the recorded signal still displayed an unsatisfactory appearance (Figure 2A).

Optimal performance was ultimately achieved through an additional stabilization step. This involved performing 100 cyclic voltammetric scans in a 0.1 M phosphate buffer solution (pH 7) over the potential range from 0 to +0.5 V (vs. Ag/AgCl, 3 M KCl) at a scan rate of 100 mV·s^−1^. As shown in Figure 2A, following this procedure, the potassium ferrocyanide signal exhibited high repeatability with an appearance consistent with a diffusion-controlled electron transfer process with good reversibility. To assess the reproducibility of the fabrication approach and the activation/cleaning treatment, 10 independent 3D-PtLEs were fabricated following the same procedure, using different production batches prepared on different days and subsequently tested. The electrochemical responses of the resulting electrodes showed good electrode-to-electrode consistency, confirming the reliability and robustness of the proposed manufacturing strategy. In particular, the electrodes exhibited a consistent peak-to-peak separation (95 mV), with a relative standard deviation (RSD) of 4.8%, demonstrating the high reproducibility of the fabrication and surface treatment procedures. The progressive improvement in the electrochemical behavior of 3D-PtLEs throughout these cleaning and activation steps is illustrated in the inset of Figure 2. This sequence may facilitate the removal/reduction of surface oxide species formed during acidic activation, contributing to platinum surface stabilization [50,51], which is critical for ensuring reproducible analytical results and robust device performance. In addition, repeated cycling in phosphate buffer may further promote reorganization of the platinum–electrolyte interface through stabilization of the electrical double layer and possible changes in the adsorption equilibrium of electrolyte species, such as phosphate ions. These combined processes likely contribute to the establishment of a stable and reproducible voltammetric response.

The voltammetric profile recorded post-surface cleaning using K_4_[Fe(CN)_6_] as a redox probe with a scan rate of 50 mV·s^−1^ exhibited a clear and well-defined shape. The peak-to-peak separation closely resembled that observed for a disc electrode (85 mV) following mechanical cleaning. Notably, for the disk electrode, the voltammetric profile of potassium hexacyanoferrate(II) showed no significant changes when subjected to the same treatment after the mechanical cleaning cycle.

Typical EC investigations and characterization, as reported in the Supplementary Materials, were then performed. A linear relationship between the peak current and the square root of the scan rate was observed for the K_4_[Fe(CN)_6_] redox probe, described by the equation y(µA) = 20.85x(V·s^−1^)^1/2^ − 0.22 ($R^{2}$ = 0.998), indicating a diffusion-controlled electrochemical process (Figure S1A). Subsequently, cyclic voltammograms were recorded at pretreated 3D-PtLEs using the electrochemical probe [Ru(NH_3_)_6_]^3+^. Different scan rates were employed to investigate electron transfer dynamics through the electrolyte (Figure S1). Well-defined profiles were recorded with a peak-to-peak separation (∆Ep) of 90 mV at a scan rate of 50 mV·s^−1^, closely resembling that at a disc electrode post-mechanical cleaning (80 mV). In these tests as well, a linear relationship between the peak current and the square root of the scan rate was observed (*y*(µA) = −89.38*x*(V·s^−1^)^1/2^ − 5.46; R^2^ = 0.998). These observations, characteristic of diffusion-controlled electrochemical processes, allow the slope of the regression plot to be linked to the electrode’s electrochemical area via the Randles–Sevcik equation for quasi-reversible processes, as detailed in the Supplementary Materials [52,53,54,55]. Scan rate studies also enabled the determination of the heterogeneous electron transfer rate constant (*k*^0^) using the Nicholson approach [56]. At 3D-PtLEs, *k*^0^ values of (4.51 ± 0.7)·10^−3^ cm·s^−1^ and (6.19 ± 0.9)·10^−3^ cm·s^−1^ were noted. Meanwhile, at platinum disc electrodes, these values were (6.5 ± 0.6)·10^−3^ cm·s^−1^ and (9.6 ± 1.2)·10^−3^ cm·s^−1^ during experiments with K_4_[Fe(CN)_6_] and [Ru(NH_3_)_6_]Cl_3_, respectively.

### 3.2. Detection of H_2_O_2_ by Linear Sweep Voltammetry

The present study is not intended to provide an exhaustive selectivity assessment typical of fully developed analytical sensors, but rather to demonstrate the Pt-like electrochemical behavior of the proposed platinum leaf electrodes. In this context, hydrogen peroxide was used as a benchmark redox probe due to its well-established sensitivity to platinum surface properties and its widespread use for evaluating platinum electrocatalytic activity. In addition, hydrogen peroxide, known for its strong oxidizing properties, is extensively utilized for surface sanitization in medical and food-related settings. However, residual traces on surfaces present potential health risks, including skin irritation and damage to mucous membranes.

Before analyzing real samples, the device was calibrated using standard solutions of H_2_O_2_ prepared in a 0.1 M phosphate buffer solution at pH 7. Linear sweep voltammetry (LSV) was applied for this purpose, scanning from +0.3 V to +1.1 V at a rate of 100 mV·s^−1^. Each test employed an 80 µL sample in drop mode, utilizing the device’s built-in platinum counter electrode and an external Ag/AgCl (3 M KCl) reference electrode.

**Figure 3:**
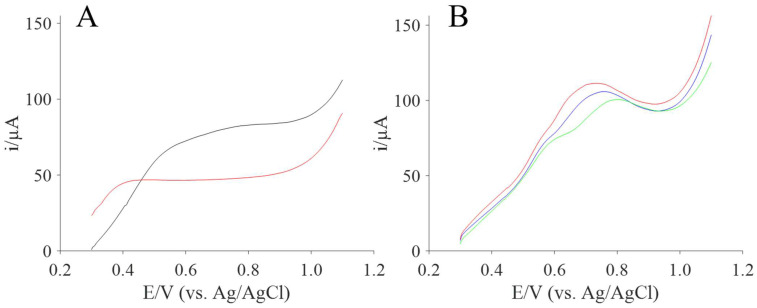
(**A**) Linear sweep voltammograms of 1 mM H_2_O_2_ in 0.1 M phosphate buffer (pH 7) recorded at pretreated 3D-PtLE during the first (black line) and second (red line) scans. (**B**) Linear sweep voltammograms of 1 mM of H_2_O_2_ in 0.1 M phosphate buffer (pH 7) recorded at 3D-PtLE after conditioning at −0.4 V for 60 s at first (red line), second (blue line) and third (green line) scan.

As illustrated in Figure 3A, immediate device testing after cleaning failed to detect any discernible signal corresponding to the analyte. To address this issue, an electrochemical pretreatment step was also introduced in this case. This step involved in situ activation at a constant potential of −0.4 V for 60 s within the same analytical solution. Its purpose was the reduction of platinum oxides on the electrode’s surface to restore its metallic state [57]. However, despite the generation of an anodic signal around +700 mV, the peak current exhibited poor reproducibility over time due to the electroadsorption of intermediate species on the electrode’s active surface (Figure 3B) [58].

**Figure 4:**
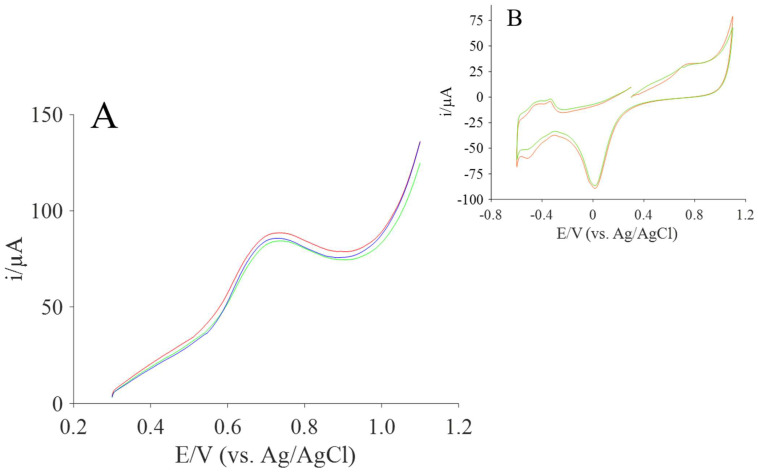
(**A**) Repeated linear sweep voltammograms of 1 mM H_2_O_2_ in 0.1 M phosphate buffer (pH 7) recorded after conditioning at −0.4 V for 60 s and treatment with 15 cyclic voltammetries in the same sample (red, green and blue lines). Inset (**B**): Cyclic voltammograms background recorded on phosphate buffer 0.1 M (pH 7) recorded at 3D-PtLE conditioned at −0.4 V for 60 s (green line) and after treatment with 15 cyclic voltammetries in phosphate buffer 0.1 M (pH 7) vs. an external Ag/AgCl, 3M KCl (orange line). Scan rate 50 mV·s^−1^.

To improve signal stability and resolution, a further stabilization phase was conducted between the activation and the analytical scan. This included 15 voltammetric cycles within a potential range of +0.1 to +0.5 V at a scan rate of 100 mV·s^−1^. This potential range was selected to remain below the H_2_O_2_ oxidation potential. This protocol generated a well-defined oxidation peak at +710 mV, as shown in Figure 4A. The intra-device repeatability showed relative standard deviations (RSD%) of 4.3 and 3.1 *(n* = 10) for peak current and peak potential, respectively. In terms of inter-device reproducibility, RSD% values were recorded as 7.3 for peak current and 4.3 for peak potential (*n* = 7).

The inset in Figure 4, which reports cyclic voltammograms in the background from −0.6 to +1.1 V, confirms that this step effectively stabilized the electrode surface and eliminated adsorbed intermediates.

**Figure 5:**
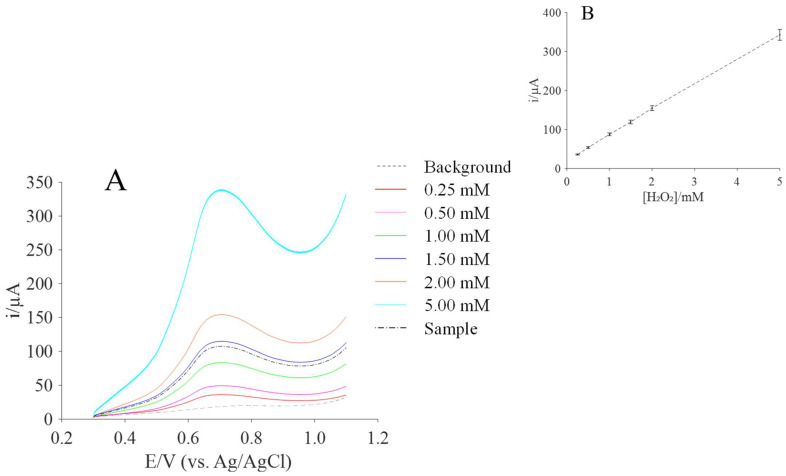
(**A**) Linear sweep voltammetry responses recorded at a 3D-PtLE in 0.1 M phosphate buffer (pH 7) (dashed line) to which increasing concentrations of H_2_O_2_ were added. The LSV response of the real sample is reported (dash-dotted line). Inset (**B**): Calibration plots of the current peak at +710 mV.

A calibration plot was subsequently constructed, which is reported in Figure 5. Within the concentration range from 0.25 to 5.0 mM, the anodic peak displayed a consistent linear response, which was found to be *y*(µA) = 64.35 *C_H2O2_*(mM) + 22.35, with an R^2^ value of 0.999. The tests were performed in triplicate for each concentration and showed good repeatability for all the concentrations evaluated, with RSD% values ranging from 3.2% to 4.1%. The detection limit, determined using the formula 3σ/S (where σ is the background noise standard deviation and S is the calibration plot sensitivity), was calculated to be 67 µM.

Finally, the device’s durability was tested through ferrocyanide voltammetric scans performed every 30 min over a total duration of 3 h. At the conclusion of these trials, RSD% values were 4.2 and 4.5 for peak current and peak potential, respectively, demonstrating that the device displayed good performance well beyond the time required to construct a calibration plot, thus confirming its long-term stability.

A comparative overview of recently reported platinum-based electrochemical sensors for hydrogen peroxide determination is reported in Table 1 [59,60,61,62], allowing the evaluation of the proposed 3D-PtLEs considering both analytical performance and, above all, the simplicity and effectiveness of the fabrication approach.

The proposed device was not specifically developed as a dedicated hydrogen peroxide sensor; rather, H_2_O_2_ was selected as a model analyte to assess and demonstrate the electrocatalytic properties of the integrated platinum leaf. Therefore, the comparison with previously reported Pt-based H_2_O_2_ sensors should be considered primarily in terms of the electrode fabrication strategy and preservation of Pt electrocatalytic activity rather than as a direct comparison of sensor optimization. Notably, most literature reports rely on amperometric detection, which generally provides higher sensitivity, whereas the proposed 3D-PtLE was evaluated by cyclic voltammetry, a simpler and less sensitive approach. Despite this difference, the device showed good analytical performance, confirming the preservation of the intrinsic electrocatalytic activity of platinum after integration into the 3D-printed platform, while being obtained through a simple, low-step fabrication strategy that avoids particular ink formulation, complex nanomaterial synthesis and surface modification procedures.

### 3.3. Real Sample Analysis

To demonstrate the practical applicability of the proposed 3D-PtLE, we applied it to the quantification of hydrogen peroxide (H_2_O_2_) in washing water prepared from sodium percarbonate (2Na_2_CO_3_·3H_2_O_2_) tablets. The cleaning effect of these products relies on the thermal breakdown of percarbonate in hot water, yielding H_2_O_2_ according to the following reaction [63]:

2Na_2_CO_3_·3H_2_O_2_ → 2Na_2_CO_3_ + 3H_2_O_2_

To minimize matrix interference and ensure that the concentration remained within the linear range of the calibration, sample solutions were cooled and then diluted at a 1:10 ratio (*v*/*v*) using a 0.1 M phosphate buffer with a pH of 7.

Figure 5A (dash–dotted line) shows a well-defined and reproducible oxidation peak during the analysis, confirming the consistent catalytic activity of platinum toward the electro-oxidation of hydrogen peroxide. This electrocatalytic response is maintained even in the presence of moderate concentrations of surfactants. No change in the electrochemical behavior was observed that could be attributed to the presence of potential interfering species, in agreement with the sample composition declared by the manufacturer. Moreover, it is important to underline that no visible delamination, deformation, or deterioration of the platinum layer was observed after repeated handling, cleaning procedures, and electrochemical testing, suggesting good mechanical robustness of the proposed configuration.

The concentration of H_2_O_2_ in the original solution was calculated to be 306 ppm (9 mM), with a relative standard deviation (RSD%) of 7.3 (n = 5). The analytical results were also validated against those obtained by the classical potassium permanganate titration method [64], showing recovery values for the spiked samples in the range of (98 ± 6%), with satisfactory relative standard deviations (RSDs).

These findings highlight the device’s reliability for monitoring H_2_O_2_ in both domestic and industrial environments with complex sample matrices.

No further interference studies in more complex matrices were performed, as the main objective of this work was not the development of a fully optimized analytical sensor, but rather the demonstration that platinum leaf materials combined with a 3D-printing fabrication approach can be effectively employed for the realization of functional electrochemical platforms. Future studies will focus on a more extensive evaluation of selectivity and matrix effects for specific analytical applications.

## 4. Conclusions

This research introduces an innovative methodology for fabricating electrochemical devices by integrating the adhesive property of polycaprolactone (PCL), the flexibility of 3D printing, and the purity of platinum leaf as an electrode material (3D-PtLEs), fully adhering to the principles of Green Analytical Chemistry (GAC) and Democratic Analytical Chemistry (DAC). This approach significantly simplifies the production process, rendering it accessible to operators with limited technical expertise, while minimizing reliance on costly equipment and substantially reducing the use of harmful solvents.

The resulting devices exhibit an estimated low production cost of 0.40 € per unit and demonstrate high reproducibility in manufacturing. Analytical validation, achieved by detecting hydrogen peroxide in both synthetic matrices and real samples, confirms the robust analytical performance of 3D-PtLEs.

This versatility is supported by the broad commercial availability of metal leaves. The results in terms of selectivity and sensitivity can be significantly improved by modifying the electrode surface with nanomaterials deposited through electrochemical methods or by drop-casting, as demonstrated by several approaches reported in the literature [41,59,60,61,62].

In alignment with the principles of circular and sustainable economies, device manufacturing employs biodegradable polymers such as polylactic acid (PLA) and polycaprolactone (PCL). Moreover, any waste generated during the manufacturing process can be fully recovered, enabling its reuse in the fabrication of additional electrochemical sensors utilizing adhesive substrates [38,65].

As future perspectives, these electrodes could be further exploited for the development of miniaturized analytical platforms, including 3D-printed flow-injection systems. Moreover, the commercial availability of several metals in the form of thin foils, such as copper, silver, and palladium, opens the possibility of extending this fabrication strategy to the production of a broader range of 3D-MLEs, thereby widening their applicability across different electrochemical sensing and analytical fields.

## Figures and Tables

**Table 1:** Comparison of the analytical performance and fabrication strategies of platinum-based electrochemical sensors for hydrogen peroxide determination.

Electrode Configuration	Construction Approach and Complexity	EC Technique	Linear Range and LOD	Ref.
Pt screen-printed electrode	Screen printing of Pt ink on carbon-based electrodes (Medium)	Amperometry	100 to 1000 μM (0.14 μM)	[41]
Pt disc electrodes modified with MWCNTs/Pt nanohybrids	Nanohybrid synthesis and deposition on the surface of Pt electrode (Medium)	Amperometry	0.01 to 2.0 μM (0.3 μM)	[59]
Pt disk electrode covered by sonoelectrodeposited platinum hierarchical nanoflowers	Electrodeposition of Pt nanoflower (Medium)	Amperometry	10–400 μM (0.32 μM)	[60]
Self-assembling of SiO_2_/AuPt nanostructures on glassy carbon modified with 3-aminopropyl-trimethoxysilane (APTMS)	Modification of the electrode with APTMS, synthesis of the SiO_2_/AuPt and their self-assembling on the modified surface (Medium–High)	Amperometry	5.0–72,000 μM (2.6 μM)	[61]
Pt nanoparticles (PtNPs) electrochemically deposited on previously modified and activated screen-printed carbon electrodes (aSPCEs) were constructed.	Activation treatment with hydrogen peroxide followed by the electrodeposition of poly(azure A) films (PAA) and platinization into H_2_PtCl_6_ solution (Medium–High)	Amperometry	0 to 300 μM (51.6 nM)	[62]
3D-PtLEs	3D printing of PLA/PCL and direct integration of pure Pt (Low)	Cyclic Voltammetry	0.25–5 mM (67 μM)	[This work]

## Data Availability

The original contributions presented in this study are included in the article.

## Supplementary Materials

The following supporting information can be downloaded at: https://www.mdpi.com/article/10.3390/s26144528/s1, Figure S1: Cyclic voltammograms recorded at scan rates ranging from 5 to 200 mV·s^−1^ at 3D-PtLEs in 0.1 M KCl containing (A) 1 mM K_4_[Fe(CN)_6_] or (B) 1 mM [Ru(NH_3_)_6_]Cl_3_. Insets, cyclic voltammograms recorded at a mechanically polished platinum disc electrode for (C) 1 mM K_4_[Fe(CN)_6_] or (D) 1 mM [Ru(NH_3_)_6_]Cl_3_ in 0.1 M KCl at scan rate of 50 mV·s^−1^.
