# Supplementary material for: Fabrication of Low-Cost and Customizable Planar Electrochemical Devices Using Multi-Material 3D Printing and Platinum Leaves
Source: Sensors (Basel). 2026 Jul 16;26(14):4528. doi: 10.3390/s26144528 (PMC13419313; doi:10.3390/s26144528)
Supplement: Supplementary file 1 [file sensors-26-04528-s001.zip › sensors-4415346-supplementary.pdf]

sensors

MDPI

Article

Fabrication of Low-Cost and Customizable Planar Electrochemical Devices Using Multi-material 3D Printing and Platinum Leaves

Michele Abate, Gino Bontempelli and Nicolò Dossi *

Sustainable Analytical Instrumentation Laboratory (Sustain Lab), Department of Agricultural, Food, Environmental and Animal Science, University of Udine, via Cotonificio 108, I-33100 Udine, Italy; michele.abate@uniud.it (M.A.); gino.bontempelli@uniud.it (G.B.)

* Correspondence: nicolo.dossi@uniud.it; Tel.: +39-0432-558835; Fax: +39-0432-558803

SUPPLEMENTARY MATERIALS

The electrochemically active surface area of the 3D-PtLE was determined using the Randles–Sevcik equation for quasi-reversible electrochemical systems. The calculation was based on the slope obtained from the linear regression of the peak current as a function of the square root of the scan rate for the two redox probes $K_4[Fe(CN)_6]$ and $[Ru(NH_3)_6]Cl_3$, measured in 0.1 M KCl supporting electrolyte.

For quasi-reversible processes, the Randles–Sevcik equation is expressed as:

$$i_p^{quasi} = \pm 0.436\, nFAC \left(\frac{nFvD}{RT}\right)^{\frac{1}{2}} \tag{S1}$$

where *ip* is the peak current (A), *n* is the number of electrons transferred (*n*=1), *F* is the Faraday constant (*F*= 96485 C·mol^{-1}), *A* is the electrode area (cm^2), *C* is the concentration of the redox probe (mol·cm^{-3}), *v* is the scan rate (V·s^{-1}), *D* is the diffusion coefficient (*D* values of 6.8×10^{-6} cm^2·s^{-1} and $9.1\text{x}10^{-6}$ cm^2·s^{-1} are reported for $K_4[Fe(CN)_6]$ and $[Ru(NH_3)_6]Cl_3$, in 0.1 M KCl supporting electrolyte, respectively), *R* is the gas constant (R=8.314 J·K^{-1}·mol^{-1}) and *T* is the temperature (T=298 K) [52-55].

The electrochemically active surface area of the 3D-PtLEs, calculated using the two redox probes, corresponded to 102 ± 4% and 109 ± 5% of the apparent geometric surface area for $K_4[Fe(CN)_6]$ and $[Ru(NH_3)_6]Cl_3$, respectively, indicating that the 3D-PtLEs exhibit a highly accessible and electrochemically active surface. In contrast, the corresponding values obtained for the disc electrode were approximately 98 ±2% and 99 ±2% for $K_4[Fe(CN)_6]$ and $[Ru(NH_3)_6]Cl_3$, respectively. These results suggest a slightly higher electrochemically active surface area, and consequently a higher surface roughness, for the 3D-PtLEs compared with the disc electrode.

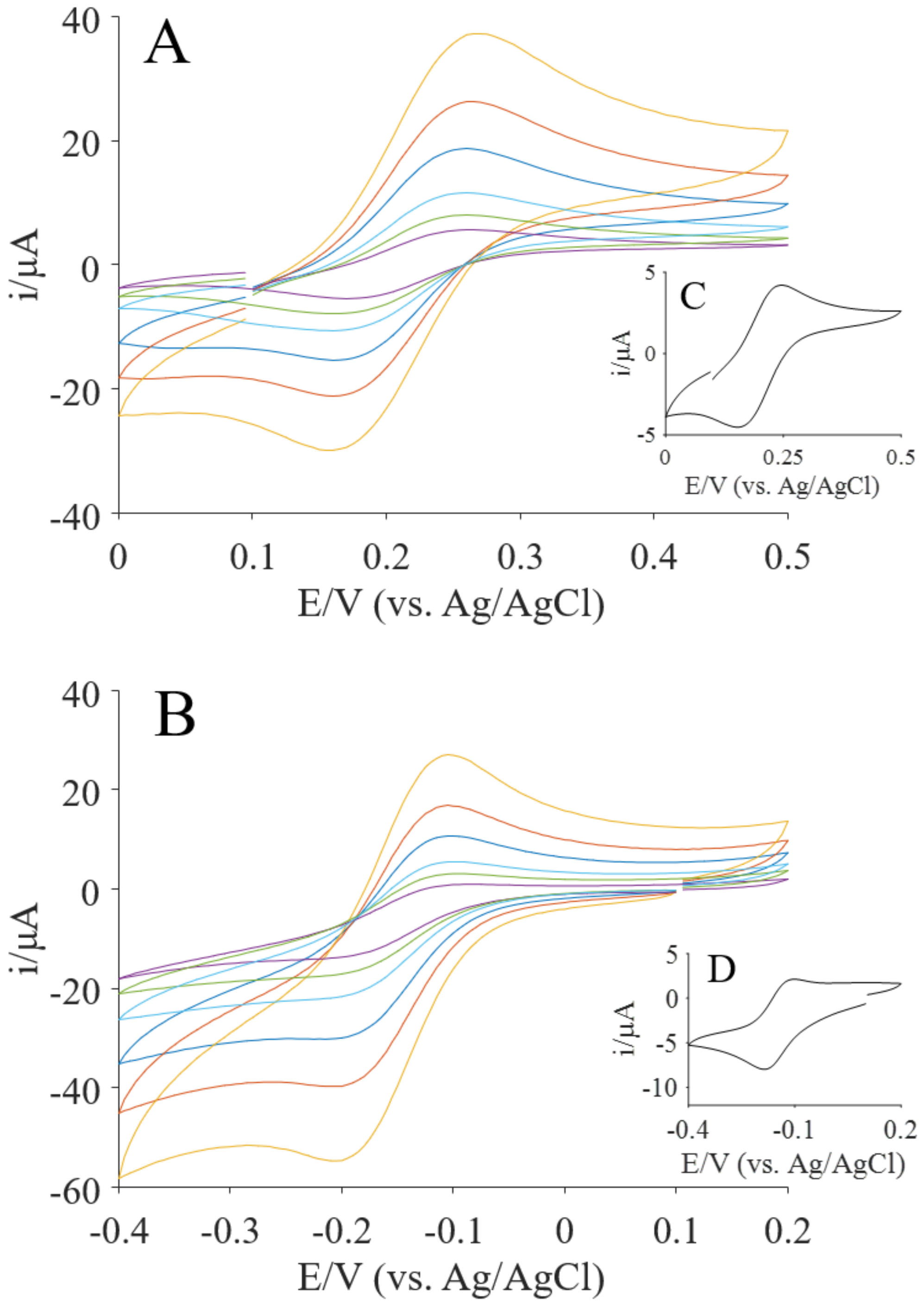

.

Figure S1. Cyclic voltammograms recorded at scan rates ranging from 5 to 200 mV·s^{-1} at 3D-PtLEs in 0.1 M KCl containing (A) 1 mM $K_4[Fe(CN)_6]$ or (B) 1 mM $[Ru(NH_3)_6]Cl_3$. Insets, cyclic voltammograms recorded at a mechanically polished platinum disc electrode for (C) 1 mM $K_4[Fe(CN)_6]$ or (D) 1 mM $[Ru(NH_3)_6]Cl_3$ in 0.1 M KCl at scan rate of 50 mV·s^{-1}.
